# Intravenous delivery of a liposomal formulation of voriconazole improves drug pharmacokinetics, tissue distribution, and enhances antifungal activity

**DOI:** 10.1080/10717544.2018.1492046

**Published:** 2018-07-25

**Authors:** Danillo F. M. C. Veloso, Naiara I. G. M. Benedetti, Renato I. Ávila, Thiago S. A. Bastos, Thaísa C. Silva, Maria R. R. Silva, Aline C. Batista, Marize C. Valadares, Eliana M. Lima

**Affiliations:** a Faculty of Pharmacy, Laboratory of Pharmaceutical Technology – FamaTec, Federal University of Goiás, Goiânia, Brazil;; b Faculty of Pharmacy, Laboratory of Celullar Toxicology and Pharmacology – FarmaTec, Federal University of Goiás, Goiânia, Brazil;; c Veterinary School, Federal University of Goiás, Goiânia, Brazil;; d Laboratory of Micology, Institute of Tropical Pathology and Public Health, Federal University of Goiás, Goiânia, Brazil;; e Laboratory of Oral Pathology, Dental School, Federal University of Goiás, Goiânia, Brazil

**Keywords:** Drug delivery, liposomal encapsulation, tissue accumulation, systemic fungal infections

## Abstract

Voriconazole (VCZ), a triazole with a large spectrum of action is one of the most recommended antifungal agents as the first line therapy against several clinically important systemic fungal infections, including those by *Candida albicans*. This antifungal has moderate water solubility and exhibits a nonlinear pharmacokinetic (PK) profile. By entrapping VCZ into liposomes, it is possible to circumvent certain downsides of the currently available product such as a reduction in the rate of its metabolization into an inactive form, avoidance of the toxicity of the sulfobutyl ether-beta-cyclodextrin (SBECD), vehicle used to increase its solubility. PKs and biodistribution of VCZ modified by encapsulation into liposomes resulted in improved antifungal activity, due to increased specificity and tissue penetration. In this work, liposomal VCZ resulted in AUC_0–24_/MIC ratio of 53.51 ± 11.12, whereas VFEND^®^ resulted in a 2.5-fold lower AUC_0–24_/MIC ratio (21.51 ± 2.88), indicating favorable antimicrobial systemic activity. VCZ accumulation in the liver and kidneys was significantly higher when the liposomal form was used. Protection of the drug from biological degradation and reduced rate of metabolism leads to a 30% reduction of AUC of the inactive metabolite voriconazole-N-oxide (VNO) when the liposomal drug was administered. Liposomal VCZ presents an alternative therapeutic platform, leading to a safe and effective treatment against systemic fungal infections.

## Introduction

The whole world needs new medicines against infectious diseases. Thanks to antimicrobials, an increase in life expectancy, especially of immunosuppressed individuals (e.g. HIV, cancer, or transplanted patients, etc.), as well as of those living in areas lacking basic sanitation infrastructure and infection prevention programs became possible (Roemer & Krysan, 2014; Rossolini et al., 2014).

The antifungal voriconazole (VCZ), a second-generation triazole with a large spectrum of action is one of the most recommended systemic antifungal agents as the first line therapy against several types of systemic mycoses, including *Candida albicans*. *C. albicans* is the most common opportunistic fungal pathogen, which may lead to life-threatening infections once the initially localized mycosis become systemic and the immune system of the host organism is compromised (Dupont, 1995; Lopes et al., 2015). VCZ exhibits a nonlinear pharmacokinetic (PK)profile due to metabolic clearance saturation (Yamada et al., 2015). In addition, VCZ is extensively metabolized in the liver, into its main circulating metabolite, voriconazole-N-oxide (VNO), which has minimal antifungal efficacy. VNO also interferes in the metabolic activity of CYP3A4 and CYP2C19, thus interfering with the metabolism of VCZ, a substrate of the aforementioned enzymes (Martinez, 2006; Theuretzbacher et al., 2006).

The solubility of VCZ in water is limited, presenting a technological issue for the development of IV formulations. To date, the only commercially available formulation of VCZ (VFEND^®^) for injection contains sulfobutyl ether-beta-cyclodextrin (SBECD) to increase drug apparent solubility in water. Similar to other cyclodextrins, SBECD can cause several clinically relevant issues, such as nephrotoxicity, hemolysis, blood vessels congestion, and hepatic and renal toxicity due to potential accumulation (Ling et al., 2016; Maertens et al., 2016; Bellmann & Smuszkiewicz, 2017; Ledoux, et al., 2017). To overcome these issues, the use of liposomes as carriers of VCZ has been explored (de Sa et al., 2015), showing clinical advantages besides eliminating the use of beta-cyclodextrins and its associated toxicity (Roffey et al., 2003; Luke et al., 2010; Kiser et al., 2015).

These advantages include targeting to infectious sites with higher local bioavailability, since the liposomes may possibly fuse to the fungal cell membrane and facilitate transference of VCZ, in an internalization mechanism already elucidated using AmBisome^®^, a liposomal formulation that encapsulates amphotericin B (Shimizu et al., 2010; Soo Hoo, 2017).

Although the PK and pharmacodynamics (PD) parameters are highly dependent on the physical-chemical characteristics of the drug, once encapsulated into liposomes, these parameters can be modulated. Different blood circulation times, tissue distribution, and the prevention of biological degradation of the antifungal might influence the efficacy of the drug (Pinto-Alphandary et al., 2000; Pattni et al., 2015). As such, these parameters must be investigated and correlated to the proposed treatment.

In this study, we prepared and characterized a novel formulation for the antifungal VCZ entrapped into liposomes (LVCZ) for intravenous delivery. In addition, PK and tissue distribution profiles were measured. *In vitro* susceptibility analysis of fungal strains, both standard and isolated in the clinic – (*C. Albicans* and *Aspergillus* sp.) was conducted as well as *in vivo* efficacy evaluation of LVCZ using a mouse model of systemic candidiasis.

## Materials and methods

### Materials

Cholesterol and soybean phosphatidylcholine (PC) were purchased from Avanti Polar Lipids (Alabaster, AL, USA). Voriconazole (VOR) was purchased from Hangzhou Dayangchem Co., Limited (Hangzhou, China). and voriconazole-N-oxide (VNO) was purchased from Genone Biotechnologies (Rio de Janeiro, RJ, Brazil) Alpha-tocopherol and Sabouraud’s dextrose agar were purchased from Sigma Aldrich (St. Louis, MO, USA). Acetonitrile was purchased from J. T. Baker (Phillipsburg, NJ, USA). Water was purified using a Milli-Q system (Millipore, Billerica, MA, USA) with a 0.22 µm pore end-filter. All other chemicals and reagents were of analytical grade or superior.

### Fungal strains and culture conditions

For the *in vitro* susceptibility assay, a total of eight fungal strains were tested as following: two American Type Culture Collection (ATCC) strains, *C. albicans* ATCC 90028 and *Candida parapsilosis* ATCC 22019, and six clinical isolates, including two *C. albicans* (77 and 11 U) and four *Aspergillus* sp. (L, V1F2, V2, and V1F1) strains. The clinical isolates were obtained from the fungal library of the Laboratory of Mycology from Federal University of Goiás and originated from samples of patients with onychomycosis and hematologic malignancies from works previously performed at the Hospital das Clínicas da Universidade Federal de Goiás and Hospital Araújo Jorge/Associação de Combate ao Câncer em Goiás (ACCG) of Goiânia-GO. *C. albicans* ATCC 90028 strain was used to establish the mouse model of systemic candidiasis. All strains were aerobically cultured on Sabouraud agar and maintained on Sabouraud 4%-glucose agar at 37 °C overnight before the assay.

### Animal care

The *in vivo* studies were carried out on Balb/c male mice (age: 7–8 weeks; weight: 25–30 g), obtained from the Animal Facilities of the Institute of Tropical Pathology and Public Health of Federal University of Goiás (Goiânia, Brazil). Before beginning the experiments, the animals were acclimatized for a week in the laboratory. Considering that animal experimentation in infectious diseases research remains essential to understand the fundamental mechanisms of antifungal drugs as well as to meet regulatory requirements, the *in vivo* analyses conducted in this study were focused on animal welfare. The experimental design considered the minimum number of animals without reducing the scientific integrity of data generated. In additions, procedures such as reduced total amount of blood collected, small volume of injections, anesthesia, and analgesia were observed.

Animals were kept under constant environmental conditions with light-dark (12:12 h) cycles and controlled temperature (23 ± 2 °C). Water and food (standard granulated chow) were provided *ad libitum*. At the end of each experiment, the mice were anesthetized with a solution containing xylazine (10 mg/kg) and ketamine hydrochloride (100 mg/kg) administered intraperitoneally and euthanized by cervical dislocation (Hubrecht & Kirkwood, 2010). The procedures and protocols were reviewed and approved by the Research Ethics Committee of the Federal University of Goiás (UFG N° 108/2014 and 095/2016).

### Preparation and characterization of the liposomal voriconazole for intravenous delivery

A liposomal formulation was prepared by lipid-film hydration followed by extrusion. PC, cholesterol, VCZ, and alpha-tocopherol, were dissolved in chloroform (1.0:0.5:0.11:0.05 molar ratio PC:cholesterol:VCZ:alpha-tocopherol, respectively). Solvent was evaporated at 25 °C and 200 rpm under reduced pressure leading to the formation of a thin layer on the inner side of a round-bottomed flask. Lipid film was hydrated with water for a final volume reaching the concentration of 2 mg/mL of VCZ. Hydrated vesicles were reduced in size by extrusion through 200-nm-pore polycarbonate filters followed by 100-nm-pore filters, in a Lipex^®^ Extruder device (Northern Lipids Inc., Burnaby, Canada) under nitrogen pressure.

Formulations were lyophilized using sucrose as cryoprotectant (4:1 of molar ratio sucrose:PC) and reconstituted in sterile saline immediately before use. Particle size and polydispersity index (PdI) were evaluated by dynamic light scattering (DLS) in a Zetasizer Nano S (Malvern Instruments, Worcestershire, UK) and the morphology by Transmission Electron Microscopy (TEM) examined in a Jeol JEM-2100 Electron Microscope (Jeol, Tokyo, Japan) operating at 100 kV, following negative staining. Drug-free liposomes were prepared as controls.

VCZ was quantified by High Performance Liquid Chromatography (HPLC) with UV detection at 255 nm. Zeta potential was assessed *via* electrophoretic mobility in a ZetaPlus (Brookhaven Instruments, Holtsville, NY, USA). Liposome Entrapment Efficiency (EE%) was determined after ultrafiltration (Vivaspin^®^ 20 kDa membrane, SIGMA, Gillingham, UK) by the following equation:

$$EE\%\;=\frac{TD\;–\;FD}{TD}\times 100$$

where TD is the total drug content of the formulation and FD is the non-encapsulated drug (free drug, filtered through the ultrafiltration device).

### *In vitro* antifungal activity of LVCZ against *Candida* sp. and *Aspergillus* sp. yeasts

*In vitro* susceptibility of yeast and filamentous fungi was performed using the microdilution method in broth following M27-A3, M27-S4 (2008, 2012), and M38-A2 (2008), proposed by the Clinical and Laboratory Standards Institute (CLSI). All the strains used were diluted in saline at the concentration of 0.5−2.5 × 10^3^ and 0.4−5×10^4^ CFU/mL for *Candida* and *Aspergillus*, respectively. VFEND^®^ and LVCZ were diluted in the range of 0.0312−32 μg/mL.

Plates were incubated at 35 °C for 48 h for Candida species and for 72 h for *Aspergillus* species.

Minimal inhibitory concentration (MIC) was determined as the lower concentration of the compound capable of inhibiting the total growth of the microorganism. The experiment was performed in triplicate, and *C. parapsilosis* ATCC 22019 was used as control of MIC, according to CLSI 2008 (John, 2008).

For minimum fungicidal concentration (MFC) determination, 10 μL of antifungal MIC and four immediately higher concentrations were seeded in Petri dishes containing Sabouraud dextrose agar (SDA). Plates were incubated at 35 °C for 48 h for Candida species and for 72 h for *Aspergillus* species. MFC was defined as the lowest concentration of antifungal that resulted in the growth of up to two colonies, which represent the death of more than 99% of the initial inoculum (John, 2008).

### Pharmacokinetic and biodistribution studies

PK studies were carried out in healthy male Balb/c mice weighing 25–30 g (7–8 weeks of age) randomly assigned to two groups (*n* = 12 for each group) according to the formulation received. Lyophilized powders of LVCZ and VFEND^®^ were reconstituted with sterile saline to 2 mg/mL. An intravenous (I.V) bolus injection of 10 mg/kg of each formulation/group was administered *via* tail vein. Using a non-mortality model (Rauzi et al., 2017), blood samples were withdrawn from the saphenous vein at the following predetermined times: 0.25; 0.50; 1.00; 2.00; 4.00; 6.00; 16.0, and 24.0 h after intravenous administration (Diehl et al., 2001).

For the biodistribution study, mice were euthanized by cervical dislocation 4 h after receiving 10 mg/kg of LVCZ or VFEND^®^. Then, heart, spleen, kidney, lungs, liver, brain, and eyes/optic nerves, were rapidly excised, washed with ice-cold saline, blotted dry, weighed, and stored at −80 °C until analysis.

VCZ and its metabolite, VNO concentrations in whole blood and tissues were determined by HPLC using an Agilent 1200 system (Santa Clara, CA, USA), composed of a quaternary pump, an auto-sampler, a column oven, with a hyphenated API 3200 triple quadrupole mass spectrometer (MS/MS) detector (MDS-SCIEX, Concord, Ontario, Canada). Drugs and internal standard (ketoconazole) were partitioned through liquid–liquid extraction using methyl tert-butyl ether. Concentrations of VCZ and VNO in blood were determined simultaneously from blood samples collected at 0.5 and 1 h following injection.

VCZ blood concentration versus time curves were plotted, and key PK parameters were calculated by the non-compartmental analysis using Microsoft Excel^®^ V.15.12.3 and the add in: PKSolver (Zhang et al., 2010b). Results were reported as means ± standard deviation (SD).

### Mouse model of systemic candidiasis

Infection model was based on protocols previously described (Courjol et al., 2016; MacCallum, 2013). In brief, mice were first rendered neutropenic by intraperitoneal injection of cyclophosphamide (75 mg/kg/d) for 2 d. To confirm neutropenia status of each animal, blood samples were obtained by retro-orbital bleeding before (day 0) and 24 h after (day 3) cyclophosphamide treatment for hematology analysis uses ABX Micros 60 (HORIBA, Montpellier, France). On day 4, each mouse was injected intravenously by tail vein with 100 μL of saline containing *C. albicans* ATCC 90028 (1 × 10^7^ cells/mL). After 2 h of infection, the animals were treated with 150 μL of either sterile saline, VFEND^®^ or LVCZ, both at 10 mg/kg of VCZ, for 3 d. Treatment groups (*n* = 5/group) were distributed as follows: Group I – infected and treated with placebo (Placebo, control group); Group II – infected and treated with VFEND^®^ (IT-VFEND^®^ group); Group III – infected and treated with LVCZ (IT-LVCZ group). On day 7, after 24 h of treatment, mice were previously anesthetized for blood collection by cardiac puncture. After that, animals were euthanized and kidneys and liver were removed for fungal burden and histopathology analyzes. For the fungal burden evaluation, liver and kidneys sections were macerated in 3 and 5 mL of sterile saline, respectively, using a glass tissue macerator. Liver and kidneys homogenates were then diluted at 1/100 and 1/1000, respectively, and 100 μL were plated on sterile Petri dishes containing SDA. After incubation for 48 h at 35 °C, colony forming units were counted. The experimental schedule of mouse model of systemic candidiasis is shown in Supplementary data 1.

### Histopathological evaluation

Parts of liver and kidneys from each animal were kept in 10% phosphate buffered formalin (pH 7.4) at room temperature for the histopathological analysis. In brief, two sagittal macroscopic cross sections were dehydrated in graded ethanol (70–100%), cleared in xylene and then paraffin-embedded. After that, paraffin-embedded tissue sections of organs (5 μm thickness) were obtained using a microtome (Leica RM 2155, Heidelberg, Germany). After mounting, the slides were dewaxed in xylene, hydrated using graded ethanol and stained with hematoxylin and eosin (HE) or periodic acid-Schiff (PAS). The slides were observed using a light microscope (Axio Scope A1 Carl Zeiss, Jena, Germany) using a 40× objective. The images were taken using an AxioCam MRc Carl Zeiss camera and AxioVs40 version 4.7.2.0 Carl Zeis software (Carl Zeiss, Jena, Germany).

### Statistical analysis

Data were reported as mean ± SD. Statistical analysis was performed using GraphPad Prism version 5.01 software for Windows (San Diego, CA). The intergroup variation was carried out by the Student’s *t*-test or one-way Analysis of Variance (ANOVA) followed by Dunnett’s test. *p* <.05 was considered statistically significant.

## Results

### Preparation and characterization of liposomal voriconazole for intravenous delivery

**Figure 1.**
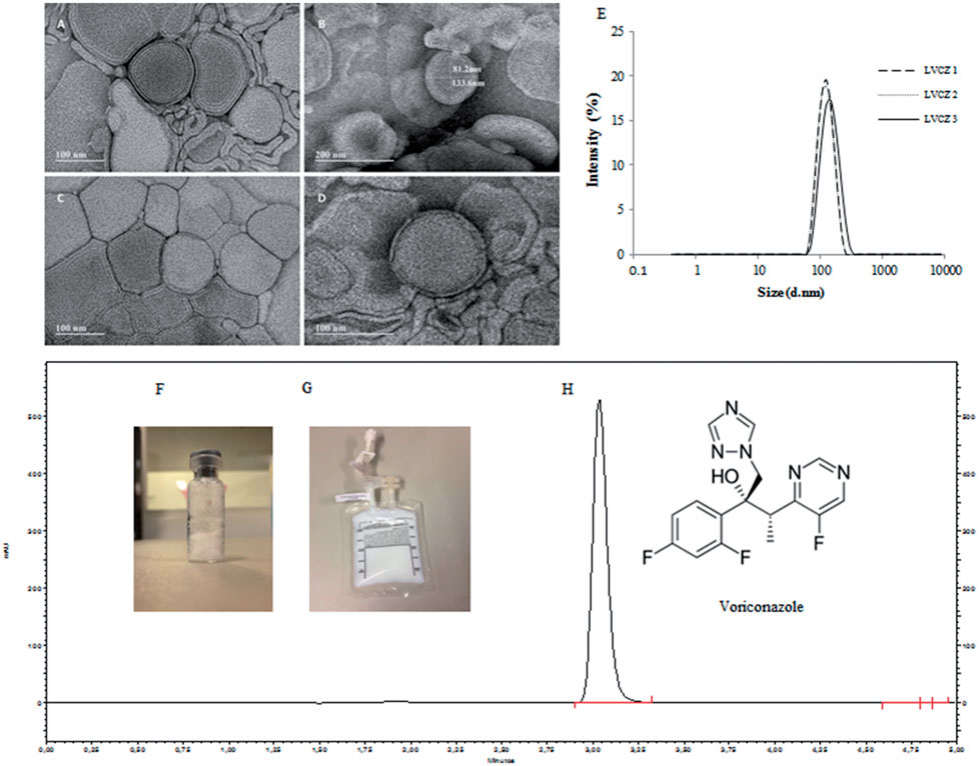
Voriconazole liposomal formulation characteristics. TEM micrograph of empty (blank) liposomes (A,B); VCZ liposomes (LVCZ) (C,D); VCZ liposomes (LVCZ) size distribution profiles by intensity obtained by DLS from three independent batches (E); freeze-dried formulation (F); redispersed freeze-dried LVCZ in a transparent infusion bag (G); and representative chromatogram from VCZ liposomal formulation at 2.0 mg/mL by HPLC with UV–VIS detector (H).

Intravenous liposomal formulation containing VCZ constituted by PC and cholesterol, herein called LVCZ, was developed for the treatment of *C. albicans* systemic infection by lipid-film hydration followed by extrusion. LVCZ exhibited a diameter of 95.3 ± 1.27 nm, very narrow size distribution (PdI =0.09 ± 0.01) and a relatively neutral charge. Entrapment efficiency was approximately 80%. Even though VCZ water solubility is low (<100 μg/mL), it is also only moderately lipophilic (log *p* ∼1.8). Drugs with intermediate partition coefficients (1.7 < log *p* < 4.0) can partition between the lipid membrane and the aqueous phase. (Gulati et al., 1998; Campbell et al., 2001; de Sa et al., 2015). TEM analysis confirmed liposomes diameter, exhibiting spherical morphology as uni- or oligo-lamellar vesicles. (more details of liposomal formulation are described in Figure 1 and also in the table of Supplementary data 2).

### *In vitro* antifungal activity of VCZ

Activities of VFEND^®^ and LVCZ against *Candida* sp. and *Aspergillus* sp. yeasts were carried out after 48 and 72 h of incubation, respectively, using the broth microdilution method. MIC and MFC values are summarized in Table 1 and show that *Candida* sp. strains were more susceptible than *Aspergillus* sp. strains for both formulations. For *Candida* sp. strains, VFEND^®^ and LVCZ have similar potency, although LVCZ triggered higher fungicide activity against *C. albicans* 77 U strain since the MFC value (0.12 μg/mL) was about 4-fold lower than that found for VFEND^®^ (0.5 μg/mL). Among *Aspergillus* sp. strains, LVCZ showed higher inhibitory/fungicide activity against *Aspergillus Flavus*. For the *A. Flavus* V2 strain, MIC and MCF values were 2- and 4-fold lower (both at 0.25 μg/mL), respectively, than those found for VFEND^®^ (MIC =0.5 μg/mL and MCF =1.0 μg/mL); while for V1F1 strain these values were 2-fold lower (MIC =0.5 μg/mL; MCF =1.0 μg/mL) in comparison to VFEND^®^ values (MIC =1.0 μg/mL and MCF =2.0 μg/mL).

**Table 1.** Minimum inhibitory (MIC) and fungicide (MFC) concentrations (μg/mL) of voriconazole and liposomal voriconazole on *Candida* species sp. and *Aspergillus* sp.

	VFEND^®^		LVCZ	
Yeast isolated	MIC	MFC	MIC	MFC
*Candida* sp.				
* C. albicans* 77 U	0.03	0.5	0.03	0.12
* C. albicans* 11 U	0.06	0.12	0.06	0.12
* C. albicans* ATCC 90028	0.03	>0.5	0.03	>0.5
* C. parapsilosis* ATCC 22019	0.03	0.03	0.03	0.03
*Aspergillus* sp.				
* A. fumigatus* L	0.5	1	0.5	0.5
* A. fumigatus* V1F2	0.25	1	0.25	1
* A. flavus* V2	0.5	1	0.25	0.25
* A. flavus* V1F1	1	2	0.5	1

ATCC: American Type Culture Collection; the designations: (77U, 11U, L, V1F2, V2, and V1F1) were used to name clinical isolates originated patients samples.

### Pharmacokinetic and tissue distribution

**Figure 2.**
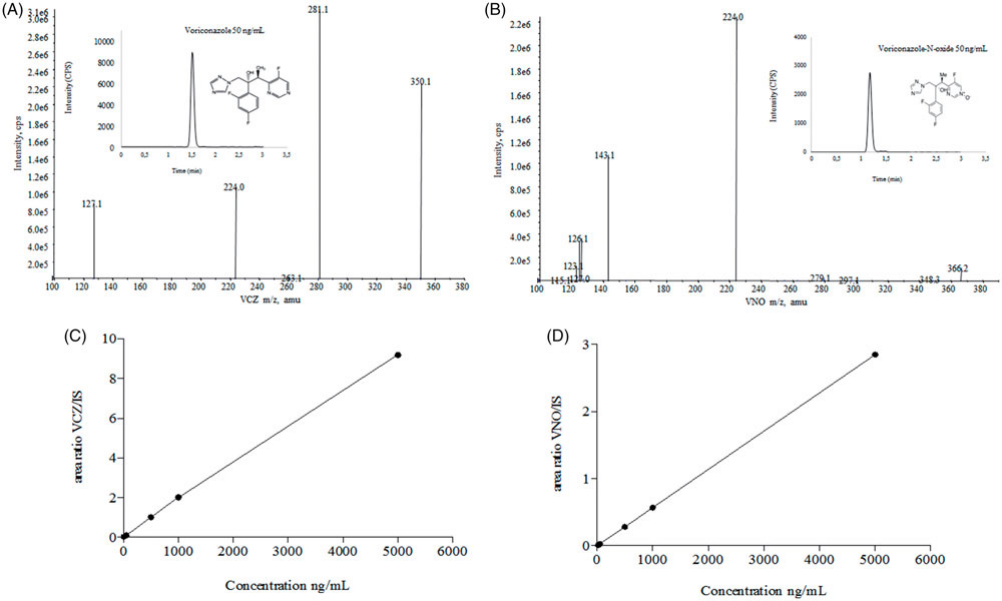
HPLC-MS/MS and analytical conditions. Full scan mass spectrum of (A) voriconazole and (B) voriconazole N-oxide by electrospray ionization in positive mode of the transitions of m/z 350.2/127.0 and m/z 366.2/224.2, respectively. Chromatographic profiles obtained under following conditions: ACE C18 column (100 × 4.6 mm ×5 µm), at 20 °C; mobile phase consisted of formic acid at 0.025% + ammonium acetate 2 mM and acetonitrile (25:75 v/v), flow rate of 1.0 mL/min. Run time was 2.0 min, and the injection volume was 5.0 µL, with an elution time for VCZ and VNO of 1.43 and 1.19 min, respectively. Calibration curves, ranging 5–5000 and 20–5000 ng/mL for VCZ (C) and VNO (D), respectively.

The bioanalytical quantitation method by HPLC-MS/MS was sensitive and selective enough to allow the simultaneous quantification of the antifungal VCZ, and its inactive metabolite VNO, even from tissue homogenates or in the whole blood samples from the animals. Chromatograms, mass spectra, and calibration curves of the above-mentioned substances are given in Figure 2.

Differences in PK parameters of VCZ from both formulations were observed. When compared to VFEND^®^, liposomes reduced VCZ clearance by half; increased AUC_0–24_ by a 2.5-fold and reduced the drug volume of distribution. Main PK parameters and data for predicting the magnitude of the best PK/PD index, which were calculated by the ratio AUC_0–24_/MIC and C_max_/MIC also are shown in Table 2.

**Table 2.** Pharmacokinetic and pharmacodynamic parameters after 10 mg/kg IV administration of two different voriconazole formulations to Balb/c mice.

			Mean ± SD	
Parameter	Rule/equation	unit	LVCZ	VFEND^®^
T_max_	observed	h	0.25 ± 0.00	0.25 ± 0.00
C_max_	observed	μg/mL	1.23 ± 0.28	0.61 ± 0.15
C_0_	extrapoled	μg/mL	1.16 ± 0.38	1.01 ± 0.39
AUC_0–24_	trapezoids	μg/mL*h	4.86 ± 1.01	1.96 ± 0.30
Cl	Dose/AUC	mL/h	52.75 ± 8.88	100.01 ± 20.14
Vd	IV dose/C_0_	mL	230.18 ± 53.61	314.18 ± 106.24
AUC_0–24_/MIC	ratio	–	53.51 ± 11.12	21.51 ± 2.88
C_max_/MIC	ratio	–	13.49 ± 31.3	6.73 ± 1.47
T > MIC	observed	h	∼12	∼8

T_max_: time to reach C_max_; C_max_: maximum plasma concentration; C_0_: estimated initial (zero-time) drug concentration in blood; AUC_0–24_: blood levels of intravenously injected VCZ versus time (0–24 h); Cl: clearance; Vd*:* volume of distribution; IV: intravenous. Pharmacodynamic parameters calculed, considering estimated voriconazole unbound fraction: AUC_0–24_/MIC: ratio between unbound fraction of voriconazole (33% of total AUC_0–24_) and MIC; C_max_/MIC: ratio between unbound fraction of voriconazole (33% of total C_max_) and MIC of *C. albicans*; T > MIC: duration of time that the voriconazole blood concentration exceeds the MIC; MIC: minimum inhibitory concentration

**Figure 3.**
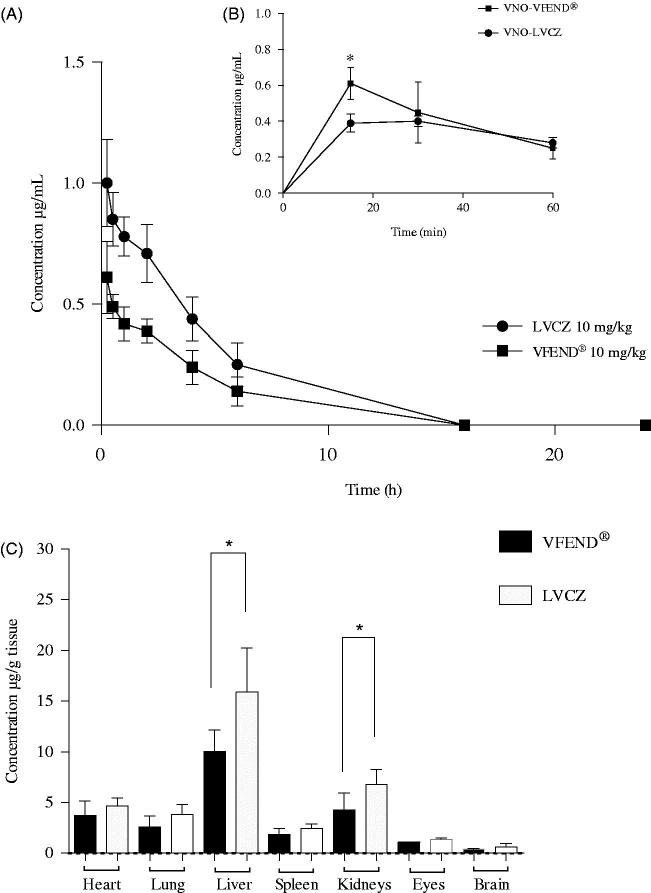
Pharmacokinetic and biodistribution profile of voriconazole from two different formulations. (A) Blood levels of intravenously injected voriconazole versus time 0–24 h (AUC_0–24_) delivered from liposomal formulation (circles) and VFEND^®^ (squares) at 10 mg/kg. (B) Concentrations versus time of the metabolite (voriconazole-N-oxide) measured within the hour following intravenous administration of the formulations. Data are expressed as mean ± SD (*n* = 12 animals per group). (C) Tissue distribution of voriconazole 4 h after I.V. administration of liposomal formulation (white bars) and voriconazole complexed in sulfobutyl ether-beta-cyclodextrin so + dium, commercially available as VFEND^®^ (black bars). Bars represent the standard deviation (*n* = 6). (*indicates *p*<.05).

Blood concentration profiles following I.V injections of VFEND^®^ or LVCZ formulations in Balb/c mice are shown in Figure 3(A). Blood levels of VCZ delivered through liposomes was higher than VFEND^®^ at any time point. Figure 3(B) shows that during the initial hour following administration of the formulations, the metabolism of VCZ when entrapped into liposomes is decelerated and thus slowing the formation of the inactive metabolite (VNO) by 30%. The initial blood concentration of VNO expressed as the AUC_0–1 h_ was 0.49 µg/mL.h for VFEND^®^ and 0.35 µg/mL.h for LVCZ.

In order to avoid bias caused by the occurrence of hysteresis (disagreement between values of tissue distribution, determined only by approximation of antifungal blood concentrations obtained from PK), the quantification of VCZ in several tissues was performed.

Tissue distribution of VCZ from both formulations was evaluated in various tissues, including several organs of the reticuloendothelial system (Figure 3(C)).

Our PK assays using healthy animals have shown that blood concentrations of VCZ remained for 12 h above the MIC, following the administration of 10 mg/kg of the liposomal formulation. Conversely, VFEND^®^ was able to maintain concentrations above MIC for only 8 h after a single bolus dose.

### *In vivo* antifungal activity of LVCZ using a mouse model of systemic candidiasis

**Figure 4.**
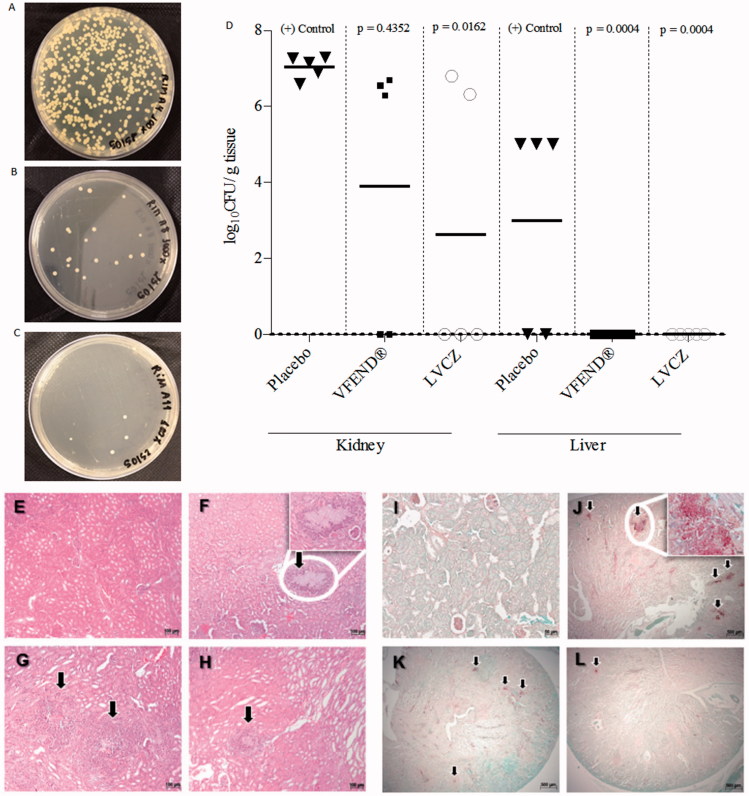
Fungal growth of *C. albicans* from kidneys of imunossupressed and infected mice treated with placebo (A), VFEND^®^ (B) and LVCZ (C). (D) Effects of the VCZ treatments on the tissue burden of *C. albicans* in infected mice and controls (*n* = 5 per group). Infection was completely resolved in the liver using VFEND^®^ or LVCZ formulation. Representative micrographs of histological analysis of H&E stained kidney sections of (E) control group, healthy Balb/C mice, (F) infected mice, placebo group, (G) infected mice, treated with VFEND^®^ and (H) infected mice, treated with LVCZ. PAS stained kidney sections showing fungal invasion in (I) control group, healthy Balb/C mice, (J) infected mice, placebo group, (K) infected mice, treated with VFEND^®^ and (L) infected mice, treated with LVCZ. Inserts are higher magnification of fungal invasion areas in the kidney tissue, also indicated by black arrows.

Antifungal activity measured by fungal burden demonstrated that treatment with LVCZ reduced significantly the number of fungal cells in the kidneys. LVCZ and VFEND^®^ formulations showed inhibitory activity against fungal growth (Figure 4(A,B)) when compared with the positive control (Figure 4(C), placebo group). Kidney fungal burden for each group expressed numerically was 2.62 ± 3.60 and 3.91 ± 3.57 log_10_ CFU/g for LVCZ and VFEND^®^, respectively, after 3d of treatment. Infected animals that have not undergone any treatment (positive control group) exhibited 7.03 ± 0.29 log_10_ CFU/g. Livers of the animals from both groups were cleared from fungal cells (Figure 4(D)), while livers of animals from the control group had a 2.96 ± 2.71 log_10_ CFU/g.

All animals presented different degrees of weight loss (Supplementary data 3(B)), strikingly evident for the placebo group.

### Histopathological evaluation

Figure 4 shows representative HE (4E–H) and PAS (4I–L) staining photomicrographs of kidney sections of healthy and *C. albicans*-infected mice following treatment with VFEND^®^ or LVCZ for 3 d. In contrast to the normal kidney structure found in the healthy control group, infected mice from the placebo group showed extensive fungal foci with necrotic tissue damage in the areas around the infection. On the other hand, treated animals, particularly with LVCZ, showed minimal damages, with inflammatory response predominant due to neutrophil infiltration.

## Discussion

Understanding the failures in the therapies that use drugs widely employed for the treatment of fungal systemic infections open new perspectives for the development of drug delivery systems. As such, the design of a liposomal formulation potentially able to overcome pitfalls of the current treatments used for *C. albicans* infections seems to be a viable alternative.

Inherent flaws in therapies that use VCZ (e.g. accelerated VCZ metabolism, low selectivity of the antimicrobial in its complexed form to the site of infection), may leave gaps for microbial resistance (Graybill et al., 2003; Voltan et al., 2016). For this reason, new formulations with higher specificities or capabilities to overcome these issues are in demand. When developing a new formulation, in particular, new drug delivery systems such as the liposomal VCZ used in this study, the knowledge of the PK s and biodistribution is crucial to predict *in vivo* antimicrobial activity. Susceptibility data against fungal infections such as *C. albicans*, one of the most prevalent fungal pathogen in lethal bloodstream infections of immunocompromised patients (Andes, 2006; Seneviratne et al., 2011; Das et al., 2015) are also crucial information.

*In vitro* antifungal effect shows good antifungal efficacy, both in commercial and liposomal formulation. Meanwhile, LVCZ showed superior performance than VFEND, suggesting that the liposomal formulation allowed superiority in inhibition of fungal growth even at lower VCZ concentrations. Which may be attributed to the effect of liposomes to across fungal cell wall, reach and enter in the cell membrane of the microorganism and release high quantities of VCZ also from the inside out (Shimizu et al., 2010; Ling et al., 2016; Soo Hoo 2017).

PK and biodistribution results indicated a higher renal and hepatic drug accumulation, supporting additional mechanisms of the LVCZ to enhance antifungal activity (Peer et al., 2007; Das et al., 2015). Tissue inflammation caused by *C. albicans* colonization, results in a remarkable change in blood vessels permeability forming fenestrations and allowing liposomes to accumulate by passive targeting (Maeda, 1996; Bazak et al., 2014; Sercombe et al., 2015). This accumulation, indicated by a higher drug concentration, was shown in liver and kidneys (important target organs of the colonization of this fungal species) (Calderone & Fonzi, 2001; Lionakis et al., 2011; Hohl, 2014). The release of the antifungal at these sites of infection, might contribute to an enhanced VCZ action, also reducing its toxicity to non-target tissues (Groll et al., 2000).

All PK/PD parameters of the antimicrobial were modulated by liposomal entrapment, leading to a more effective formulation. The use of liposomes as VCZ carriers enhanced all relevant parameters of the treatment against *C. albicans* infection (Zhang et al., 2010; Huh & Kwon, 2011; Merian et al., 2015).

Reduced conversion of VCZ into VNO during the metabolism phase indicates that liposomal entrapment protects the drug from metabolism in the initial stages of circulation and distribution. Decelerating the metabolism of VCZ thus slowing the formation of the inactive metabolite (VNO), might be a mechanism of improving the existing drug therapy (Peer et al., 2007; Li et al., 2017).

AUC_0–24 h_ obtained of VCZ unbound plasma protein (Roffey et al., 2003) (33% of the AUC_0–24 h_ originally obtained in the PK assay using healthy animals) remained for approximately 12 h above the MIC (i.e. a T > MIC =12 h), following the administration of 10 mg/kg of the LVCZ, ensuring the maximization of antifungal exposure and also avoiding toxicity by exposure to unnecessary high concentrations of the drug (Goodwin & Drew, 2008; Decosterd et al., 2010).

Moreover, in studies where VCZ was used in the treatment of *C. albicans*, it was well known that the fungistatic activity of the triazole is not related to its concentration, conversely, VCZ activity is time-dependent (Klepser, et al., 2000; Theuretzbacher, et al., 2006). Previous literature reports that best antifungal activity for VCZ is reached when AUC_0–24_/MIC ratio >32 (Andes, 2006). In this work, using the liposomal formulation AUC_0–24_/MIC was 53.51 ± 11.12, whereas VFEND^®^ resulted in a 2.5-fold lower AUC_0–24_/MIC ratio (21.51 ± 2.88). In clinical trials using azole antimicrobials for the treatment of invasive candidiasis, when this AUC/MIC ratio is >25, treatment success is at least 91% (Lepak & Andes, 2014).

Histopathological findings showed that, in contrast to the normal kidney framework found in healthy animals (Figure 4(E,I)), the non-treated *C. albicans*-infected mice showed extensive fungal foci with necrotic tissue damage in areas around the infection (Figure 4(F,J)). On the other hand, VCZ treated mice showed fewer alterations, along with an inflammatory response predominantly with neutrophil infiltration and significant reduction of fungal load (Figure 4(G,H,K,L)).

Histological findings support antifungal effect of both formulations, although PK/PD data indicate a certain degree of superiority of the liposomal formulation.

Despite the short post-antifungal effect (PAFE) of VCZ (0.2–0.4 h for *C. albicans*) (Manavathu et al., 1998a; Manavathu et al., 1998b; Andes, 2003; Theuretzbacher et al., 2006), liposomal formulation proved to be highly effective *in vivo*, when treating *C. albicans* infected mice. This efficacy was probably due to increased renal accumulation and superiority in the PK parameters of the antifungal when encapsulated in liposomes.

Improvements in PK s and a tissue distribution more selective to the organs most affected by the infection might increase the efficacy of LVCZ, when compared directly with VFEND^®^ in its capacity to reduce fungal burden from kidneys of leukopenic mice (Supplementary data 3(A) shows the decay of basal leukocyte levels, over cyclophosphamide dosing period, confirming leukopenia of animals).

Since the efficacy of antifungals cannot be estimated based merely on their blood concentration, it is important to correlate this with tissue distribution, which relates to the VCZ/fungal burden colocalization (“drug and bug”) (Theuretzbacher, 2007; Felton et al., 2014).

Our biodistribution results indicate that the liposome can penetrate into the tissues most affected by the infection, delivering the drug in deeper renal subcompartments, suffering less impact of the glomerular filtration and minimizing the conversion of the VCZ into its inactive metabolite (VNO). The extravasation of the liposomes to infected tissues was probably increased due the more permeable endothelium found in the infection sites (Maeda et al., 2000; Allen & Cullis, 2004; Phillips et al., 2010), achieving rapid and sustained levels of VCZ locally, contributing to a better resolution of the infection, as shown in liver and kidneys (Figure 4(E)). Avoiding suboptimal target site concentrations might enhance antifungal effects of VCZ and lead to a better reduction of the fungal burden in these tissues (Liu et al., 2002).

A high AUC/MIC correlation obtained in our PK/PD assays, suggests a more pronounced antifungal response when the liposomal formulation was used, highlighting a gain in the efficacy of the LVCZ formulation over VFEND^®^.

Nevertheless, limitations of murine models using this triazole may hinder further clinical correlation. Accelerated clearance and even genetic polymorphism in humans require thorough investigations involving different animal species or even patients of different age, gender, stage of disease, or ethnicity. Previous literature reports suggest the oral use of grapefruit concomitant with VCZ or the use of guinea pigs lineages instead of mice in order to reduce enzymatic activity that can lead to irrelevant serum VCZ concentrations due to their extremely rapid conversion to the inactive metabolite, VNO (Sugar & Liu, 2000; Eiden et al., 2010).

## Conclusions

Liposomal formulation entrapping VCZ is an alternative formulation for intravenous antifungal treatment, avoiding the use of potentially toxic adjuvants and showing significant improvements in antifungal activity to target systemic *C. albicans* infection in a murine model, compared to current therapy.

The ability of VCZ to exert fungicidal activity might be enhanced by its entrapment within liposomes. Liposomal encapsulation showed a certain degree of protection from premature metabolism, contributing to more effective antimicrobial blood concentrations, allowing for deeper and more selective penetration and accumulation into the tissues more severely affected by the pathogen.

We have successfully obtained an effective, biocompatible, biodegradable, targeted and safe antifungal liposomal formulation for VCZ. Liposomal VCZ can be used for intravenous delivery of the drug, resulting in improved PK s parameters with positive impact in the antifungal activity of VCZ.

Our contribution encourages the development of an alternative formulation to the commercially available VCZ for intravenous delivery. PK data also reinforce the evidence that, by encapsulating VCZ in liposomes, it is possible to slow down the metabolic depletion of the antifungal. Additionally, a more selective tissue accumulation may be an effective strategy to deliver the antifungal into deeper regions of organs severely affected by the fungal infection.

## Supplementary Material

Supplementary data 3

Supplementary data 2

Supplementary data 1
