# Supplementary material for: Intravenous delivery of a liposomal formulation of voriconazole improves drug pharmacokinetics, tissue distribution, and enhances antifungal activity
Source: Drug Deliv. 2018 Jul 25;25(1):1585–94. doi: 10.1080/10717544.2018.1492046 (PMC6060385; doi:10.1080/10717544.2018.1492046)
Supplement: Supplementary data 3 [file IDRD_A_1492046_SM3447.docx]

**Supplementary data 3**

A

B

Total leucocyte counts and weight loss of mice during experiment. (A) WBC counts and (B) weight loss were performed: 24 hours before (day 0), 24 hours after immunosuppression (day IV), and after infection/3 days of treatment with voriconazole (day VII).
