# Supplementary material for: Intravenous delivery of a liposomal formulation of voriconazole improves drug pharmacokinetics, tissue distribution, and enhances antifungal activity
Source: Drug Deliv. 2018 Jul 25;25(1):1585–94. doi: 10.1080/10717544.2018.1492046 (PMC6060385; doi:10.1080/10717544.2018.1492046)
Supplement: Supplementary data 2 [file IDRD_A_1492046_SM3446.docx]

Supplementary data 2: Summary liposomal formulation entrapping voriconazole (LVCZ) characteristics: size, polydispersity index, zeta potential, pH and encapsulation efficiency.

| **Formulation** | **size** | **PdI** | **Zeta potential** | **pH** | **EE%** |
| --- | --- | --- | --- | --- | --- |
|  | **(nm)** |  | **(mV)** |  |  |
| **Blank liposomes** | 124.5 ± 0.75 | 0.05±0.01 | -1.7 ± 1.6 | 6.36 ± 0,15 | - |
| **LVCZ 2.0 mg/mL** | 95.3 ± 1.27 | 0.09±0.01 | -0.32 ± 0.6 | 7.23 ± 0.03 | 79.80±0.90 |

Results express the mean ± SD (n = 3); PdI polydispersity index, EE% represents the amount (%) of encapsulated drug in relation to the total amount of drug added to the formulation (EE% = (mg encapsulated drug)/(mg drug added to formulation) × 100)
