# Supplementary material for: Intravenous delivery of a liposomal formulation of voriconazole improves drug pharmacokinetics, tissue distribution, and enhances antifungal activity
Source: Drug Deliv. 2018 Jul 25;25(1):1585–94. doi: 10.1080/10717544.2018.1492046 (PMC6060385; doi:10.1080/10717544.2018.1492046)
Supplement: Supplementary data 1 [file IDRD_A_1492046_SM3445.docx]

Supplementary data 1

Immunosuppression, inoculum and treatment schedule. Blood cell count was performed before, during and after immunosuppression, infection and treatment of the animals. For induction of leukopenia, Balb/c mice were exposed to cyclophosphamide at a dose of 75 mg/kg/day for two consecutive days. 24 hours after immunosuppression, animals received the inoculum of *C. albicans* (ATCC 90028) and, 1 hour later, the treatment was started (VCZ formulations at 10 mg/kg/day for 3 days). after receiving the last dose of the VCZ formulations (LVCZ or VFEND^®^), mice had their kidneys and liver excised for quantification of the fungal burden.
